# Higher blood aldosterone level in metabolic syndrome is independently related to adiposity and fasting plasma glucose

**DOI:** 10.1186/s12933-015-0175-6

**Published:** 2015-01-13

**Authors:** Jui-Kun Chiang, Chi-Ling Chen, Feng-Yu Tseng, Yu-Chiao Chi, Kuo-Chin Huang, Wei-Shiung Yang

**Affiliations:** Department of Family Medicine, Buddhist Dalin Tzu Chi Hospital, Chiayi, Taiwan; Graduate Institute of Clinical Medicine, College of Medicine, National Taiwan University, 7 Chun-Shan South Road, Taipei, 100 Taiwan; Graduate Institute of Epidemiology, College of Public Health, National Taiwan University, Taipei, Taiwan; Departments of Internal Medicine, National Taiwan University Hospital, Taipei, Taiwan; Family Medicine, National Taiwan University Hospital, Taipei, Taiwan

**Keywords:** Aldosterone, Metabolic syndrome, Adiponectin, Adiposity, Glucose

## Abstract

**Background:**

Hypoadiponectinemia is a well-known state associated with metabolic syndrome (MetS) and insulin resistance (IR). Recently aldosterone has been highly associated with high blood pressure, and may thus be a possible biomarker for MetS and IR. In this study, we investigate the association of aldosterone with MetS and IR, and compare it with that of adiponectin.

**Methods:**

In this cross-sectional study, we recruited 556 women receiving physical examinations at a general hospital in central Taiwan. At the time of examination, we collected data on various demographic and physical characteristics and measured blood levels of aldosterone, adiponectin and a variety of metabolic factors. Multiple linear regression analysis was performed using adiponectin or aldosterone as the dependent variables.

**Results:**

We found an inverse correlation between blood adiponectin and aldosterone (γ = −0.11, P = 0.009). Adiponectin levels were lower and aldosterone levels higher in women with MetS that those without (8.1 ± 0.4 vs. 11.5 ± 0.2 μg/mL, P < 0.001 and 691 ± 50 vs. 560 ± 11 pmol/L, P = 0.013, respectively), as they were in women with and without IR (adiponectin 10.4 ± 0.5 vs. 11.3 ± 0.2 μg/mL, P = 0.003 and aldosterone 635 ± 31 vs. 560 ± 11 pmol/L, P = 0.022). Although aldosterone was significantly related to body fat %, fasting plasma glucose and serum creatinine levels, the relationship between adiponectin and aldosterone was not obvious after adjustment in the multivariate analysis.

**Conclusion:**

Although aldosterone was related to metabolic factors, including body fat % and fasting plasma glucose in our female subjects, the relationship between aldosterone and adiponectin remains unclear.

## Background

Adiponectin has been found at lower blood levels in patients with metabolic syndrome (MetS) and its related co-morbidities, including obesity, type 2 diabetes mellitus (DM), dyslipidemia and hypertension [1-4]. In fact, hypoadiponectinemia may be one of the most important biomarkers for the MetS and obesity-induced insulin resistance (IR) [5,6]. Recently, activation of the renin-angiotensin-aldosterone system (RAAS) has been found to possibly play a role in the pathogenesis of obesity-related hypertension [7]. Two clinical trials have reported that blocking RAAS by either angiotensin converting enzyme inhibitors (ACEI) or angiotensin 2 receptor 1 blockers (ARB) can reduce the incidence of new-onset DM and the studies suggested that this reduction may have been a result of improvement in insulin sensitivity [8,9]. Treatment with ACEI or ARB has also been found to increase blood level of adiponectin in patients with hypertension [10,11].

Both animal and human studies have suggested that RAAS may play a role in MetS [12,13]. Previous several studies of people with high blood levels of aldosterone found a close assocation between primary aldosteronism and insulin resistance [14,15] as well as between lower blood adiponectin levels [16] and a higher prevalence of MetS [17,18], and suggested that aldosterone may also serve as a biomarker for MetS and IR [19,20]. Such an association has in fact been observed in two German populations [21].

The RAAS might also be a potential mediator of adiponectin activity [22]. Compared to men, women have been found to have higher blood levels of adiponectin [23] and aldosterone [24]. Despite these findings, the relationship between these two hormones in MetS and IR remain unclear. In an effort to better clarify their relationship, we surveyed 556 women in a routine health checkup and analyzed their blood levels of aldosterone in relation to MetS, IR and related co-morbidities, and compared the results with those for adiponectin.

## Methods

### Subjects

A total of 556 females who underwent a general health examination at Buddhist Dalin Tzu Chi Hospital in southern Taiwan between August 2009 and June 2012 were included in this study. Although we recruited male subjects, there were not enough to conduct a statistically powerful study of that gender number. Furthermore, because androgen has been shown to decrease adiponectin levels [25], their exclusion from the study may also be justified from this standpoint. In addition, subjects with current malignancy, infections, such as tuberculosis, or major organ failure, such as heart failure, liver cirrhosis and uremia were also excluded. Written informed consent was obtained from all subjects. This study was approved by the Institutional Review Board of the Buddhist Dalin Tzu Chi Hospital (No. B09801018-1 and B09901018). Demographic data, including age, gender, education levels, life style, and brief medical history, were collected. Anthropometric data, including body weight, body height and waist circumference, were measured. Seventy-seven subjects met the modified National Cholesterol Education Program definition of metabolic syndrome for Asians: (1) fasting glucose level > 5.55 mmol/L (100 mg/dL) or known diabetes with anti-diabetic treatment, (2) blood pressure > 130/85 mmHg or known hypertension with anti-hypertensive treatment, (3) fasting serum triglyceride level ≥1.70 mmol/L (150 mg/dL), (4) fasting HDL-C level < 1.30 mmol/L (50 mg/dL), and (5) waist circumference ≥80 cm. Body fat percentage was measured using a Body Composition Analyzer (Model TBF-410, Tanita Corporation, Tokyo, Japan). Sitting blood pressure was measured by an auto blood pressure machine (ProCare 100, GE Medical System Information Technologies, Inc., Piscataway, NJ, USA).

### Laboratory tests

The tests of the overnight fasting blood samples, including those for lipids, sugar, blood cell counts and other biochemical items, were assayed by an auto-analyzer (Sysmex XE-2100 Blood Cell Analyzer, Kobe, Japan). The human insulin ELISA kit (BioSource Europe S.A., Nivelles, Belgium) was used for the insulin assay. The homeostasis model of assessment (HOMA) was used to evaluate insulin resistance [26], which was defined as the HOMA-IR of the highest quartile (≥1.864 in this study). The human adiponectin ELISA kit (B-Bridge International, Inc., Mountain View, CA, USA) was used for adiponectin assay. Aldosterone was measured using a radioimmunoassay kit following the manufacturer’s instructions (Adaltis Italia S.P.A., Bologna, Italy).

#### Statistical analysis

The data were presented as means ± S.D. or S.E.M. Student’s *t*-test was used to test the difference of means between two groups. Pearson correlation analysis was performed to evaluate the correlation between two variables. Multiple linear regression analysis was performed using adiponectin or aldosterone as the dependent variables and the indicated factors as the independent variables. In the stepwise variable selection procedure, both the significance levels for entry and stay in the data set were set at 0.15. The variables considered in the selection procedure are listed in Table 1, and the best candidate final regression model was then identified based on knowledge derived from the literature. Goodness-of-fit measures, including the coefficient of determination *R*^2^, as well as statistical tools for regression diagnostics, such as residual analysis, detection of influential cases, and checking for multi-collinearity, were applied to discover model or data problems. A *p* value < 0.05 was considered significant. The statistical analysis was performed using STATA version 11.2 (Stata Corp LP, College Station, TX, USA) and the R 2.15.1 (Free Software Foundation, Inc., Boston, MA, USA) software.

**Table 1:** **Basic characteristics of the 556 women and their correlations with adiponectin and aldosterone**

		**Correlation with Adiponectin**		**Correlation with Aldosterone**	
**Variables**	**Mean ± ** **S.D.**	**Γ**	***P*** **=**	**Γ**	***P*** **=**
**Age (years)**	54.4 ± 9.8	0.09	**0.032**	−0.06	0.195
**Height (cm)**	155.4 ± 5.6	−0.01	0.805	−0.04	0.918
**Weight (kg)**	55.7 ± 7.8	−0.25	<0.001	0.07	0.105
**BMI (kg/m** ^**2**^ **)**	23.0 ± 3.1	−0.25	**<0.001**	0.07	0.081
**Waist (cm)**	72.8 ± 7.2	−0.27	**<0.001**	0.04	0.410
**Body fat (%)**	29.0 ± 5.9	−0.29	**<0.001**	0.19	**<0.001**
**SBP (mmHg)**	123.3 ± 19.7	0.06	0.155	0.02	0.680
**DBP (mmHg)**	71.0 ± 11.3	0.09	0.839	0.07	0.128
**AC glucose (mM)**	5.0 ± 1.0	−0.15	**<0.001**	0.20	**<0.001**
**Fasting insulin (IU/mL)**	6.3 ± 5.2	−0.10	**0.021**	0.09	**0.034**
**HOMA-IR**	1.4 ± 1.3	−0.13	**0.002**	0.15	**<0.001**
**TG (mM)**	1.2 ± 0.7	−0.25	**<0.001**	0.09	**0.037**
**T-Chol (mM)**	4.8 ± 0.9	0.04	0.365	0.13	**0.003**
**LDL-C (mM)**	3.1 ± 0.8	−0.07	0.115	0.11	**0.009**
**HDL-C (mM)**	1.5 ± 0.4	0.41	**<0.001**	−0.03	0.462
**BUN (mM)**	4.6 ± 1.5	0.04	0.389	0.12	**0.006**
**CRE (μmol/L)**	52.1 ± 14.1	0.05	0.246	0.19	**<0.001**
**UA (μmol/L)**	279.6 ± 71.4	−0.13	**0.003**	0.14	**0.001**
**Adiponectin (μg/mL)**	11.0 ± 4.9	-	-	−0.11	**0.009**
**Aldosterone (pmol/L)**	577.0 ± 280.2	−0.11	**0.009**	-	-

*Abbreviations*: *BMI* Body mass index, *SBP* Systolic blood pressure, *DBP* Diastolic blood pressure, *AC glucose* Fasting plasma glucose, *fasting insulin* Fasting serum insulin, *HOMA-IR* Insulin resistance index by homeostasis assessment model, *mM* mmol/L, *TG* Triglycerides, *T-Chol* Total cholesterol, *LDL-C* Low-density lipoprotein cholesterol, *HDL-C* High-density lipoprotein cholesterol, *BUN* Blood urea nitrogen, *CRE* Creatinine, *UA* Uric acid. Those p values < 0.05 were in bold form.

## Results

As can been seen in Table 1, a summary of basic characteristics, we recruited 556 women. One hundred twenty-nine (23.2%) had a BMI ≥ 25 kg/m^2^ and seventy-nine (14.2%) a waist circumference ≥ 80 cm. Sixty-seven (12.1%) had either a fasting plasma glucose ≥ 5.55 mmol/L (100 mg/dL) or a previous diagnosis of diabetes. Twenty-eight (5.0%) were taking anti-diabetics medications and 89 (16.0%) anti-hypertension medications. Blood adiponectin levels correlated significantly with age, body weight, body mass index (BMI), waist circumference (WC), body fat percentage (%), fasting plasma glucose and insulin, and insulin resistance index (HOMA-IR), triglycerides (TG), high-density lipoprotein cholesterol (HDL-C), and uric acids (UA) (Table 1). Plasma adiponectin levels correlated negatively with aldosterone levels (γ = −0.11, *p* = 0.009).

Aldosterone levels correlated positively only with body fat %, less that found for adiponectin, while correlation coefficients of adiponectin with BMI, WC and body fat % were quite comparable those of aldosterone (Table 1). In addition, like adiponectin, aldosterone was significantly correlated with fasting plasma glucose, fasting insulin and HOMA-IR, with magnitudes of the correlation about the same (Table 1). Although aldosterone was significantly correlated with TG, it had a much smaller γ than adiponectin. Nevertheless, it was significantly correlated with total cholesterol (T-Chol) and low-density lipoprotein cholesterol (LDL-C), but not HDL-C (Table 1). Finally, aldosterone concentrations were also correlated with uric acid, blood urea nitrogen and creatinine (Table 1).

**Figure 1:**
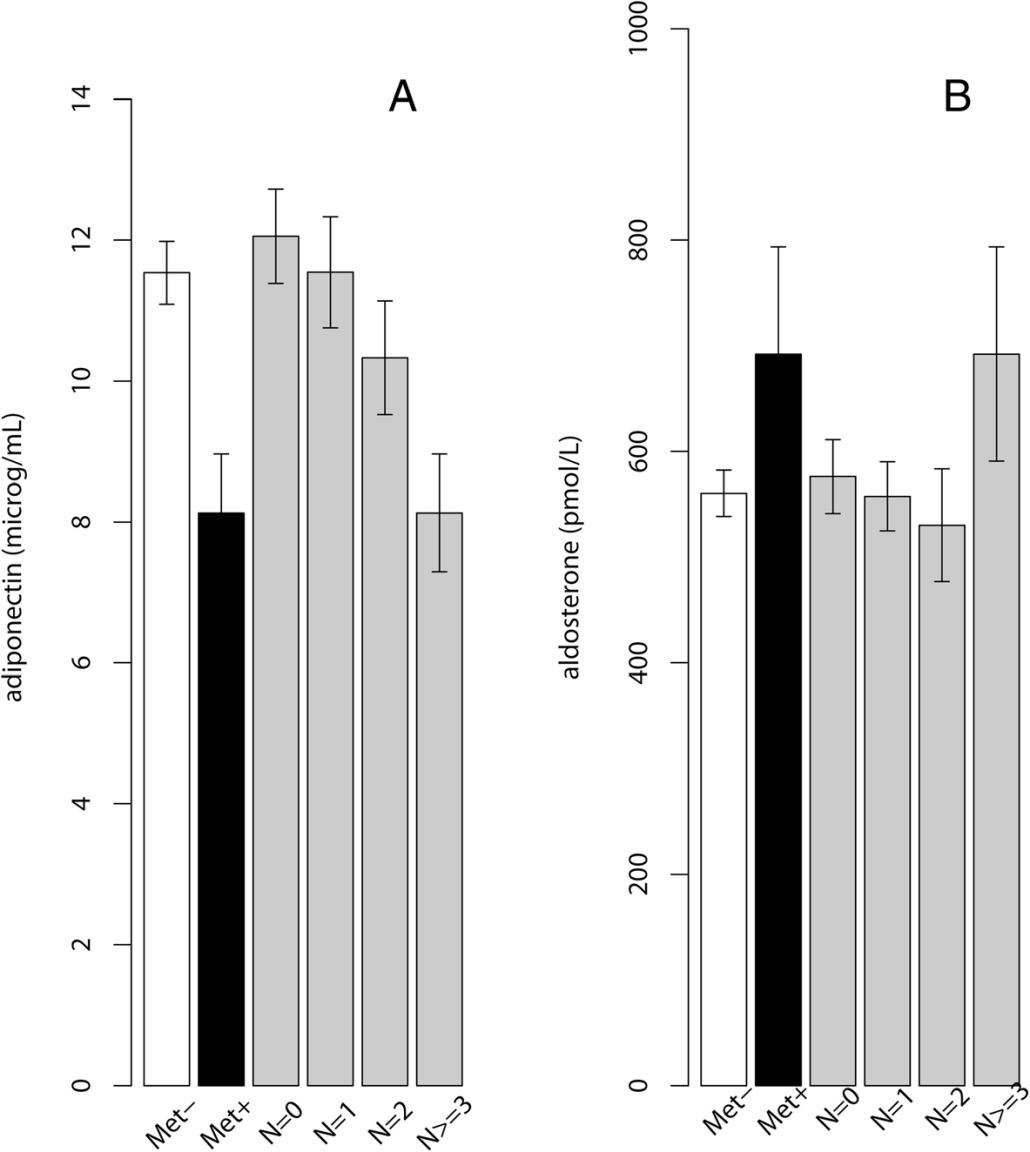
**The blood (A) adiponectin levels (Mean ± SEM) and (B) aldosterone levels (Mean ± SEM) in subjects with or without MetS or by the number of the MetS criteria met.**

The subjects were categorized into those with metabolic syndrome (MetS) and non-MetS groups (Table 2). Adiponectin levels in those with MetS (*n* = 77, 13.8%) were about around thirty percent lower (mean ± SEM: 8.1 ± 0.4 vs. 11.5 ± 0.2 μg/mL, *p <* 0.001, Figure 1A). Those meeting none, 1, 2 and 3 and more of the criteria for MetS had blood adiponectin levels of 12.1 ± 0.3, 11.5 ± 0.4, 10.2 ± 0.4 and 8.1 ± 0.4 μg/mL, respectively (mean ± SEM., *p* for trend = 0.030, Figure 1A), while they had about 19% higher levels of aldosterone (mean ± SEM: 691 ± 50 vs. 560 ± 11 pmol/L, *p* = 0.013 with unequal variances, Figure 1B). The subjects meeting none, 1, 2 and 3 and more criteria for MetS had blood aldosterone levels of 574 ± 17, 558 ± 17, 530 ± 25 and 694 ± 50 pmol/L, respectively (mean ± SEM., *p* for trend = 0.410, Figure 1B).

**Table 2:** **The adiponectin and aldosterone levels in 556 women belong to various groups**

	***n***	**Adiponectin (μg /mL)**			**Aldosterone (pmol/L)**		
**Presence of criteria**	**Yes/No**	**Yes**	**No**	**P=**	**Yes**	**No**	**P=**
**Waist** ≥ **80 cm**	**79/477**	8.9 ± 0.5	11.4 ± 0.2	**<0.001**	535 ± 28	585 ± 14	0.141
**Body fat** ≥**30%**	**215/339**	9.9 ± 0.3	11.8 ± 0.2	**<0.001**	616 ± 22	555 ± 14	**0.016**
**IFG + diabetes**	**67/489**	8.6 ± 0.5	11.4 ± 0.2	**<0.001**	671 ± 58	566 ± 11	**0.081**
**SBP > 130 or DBP > 85 mmHg or hypertension**	**219/337**	11.1 ± 0.4	11.0 ± 0.3	0.990	605 ± 22	560 ± 14	0.096
**TG** ≥ **1.7 Mm (150 mg/dL)**	**88/468**	9.0 ± 0.4	11.4 ± 0.2	**<0.001**	644 ± 33	566 ± 14	**0.035**
**HDL-C <1.3 Mm (50 mg/dL)**	**179/377**	8.9 ± 0.3	12.1 ± 0.3	**<0.001**	602 ± 25	566 ± 14	0.219
**Insulin resistance**	**139/416**	10.4 ± 0.5	11.3 ± 0.2	**0.003**	635 ± 31	560 ± 11	**0.022**
**Metabolism syndrome**	**77/479**	8.1 ± 0.4	11.5 ± 0.2	**<0.001**	691 ± 50	560 ± 11	**0.013**

*The abbreviations are the same as those in Table 1.

*The values were expressed as Means ± SEM. *Those p values < 0.05 were in bold form.

*Two missing for FAT % and one missing for HOMA-IR.

*IFG: impaired fasting glucose: AC glucose ≥ 5.55 mM.

*Insulin resistance was defined as HOMA-IR > higher quartile (1.864 in our study).

We also categorized subjects based on the various components of MetS, including central obesity, fasting glucose, triglycerides, HDL-C levels, and hypertension and insulin resistance markers (Table 2). We found a significant difference in adiponectin and aldosterone levels in centrally obese subjects only. Compared to those with impaired fasting glucose and diabetes, those with normal fasting glucose had significantly higher adiponectin levels and borderline lower aldosterone concentrations. Those with lower fasting plasma triglycerides had significantly higher adiponectin concentrations, but lower aldosterone concentrations, while those with higher HDL-C levels only had higher adiponectin levels, but not lower aldosterone levels. We found no differences in blood adiponectin and aldosterone concentrations between subjects with hypertension (SBP > 130 mmHg, DBP > 85 mmHg, or hypertension) and those with lower blood pressure. Insulin resistance was defined in subjects belonging to the highest HOMA-IR quartile. This group was found to have lower adiponectin levels but higher aldosterone levels (Table 2).

Based on our linear regression analysis, there was a negative association between adiponectin and aldosterone with borderline statistical significance after adjusting for several variables (Table 3). As expected, a larger waist circumference, impaired fasting glucose and diabetes were negatively related to blood adiponectin (Table 3). In contrast, age and HDL-C levels were positively associated with adiponectin levels. Using aldosterone as the dependent variable, we found body fat %, fasting plasma glucose and serum creatinine levels to be independently associated with blood aldosterone levels after adjustment (Table 3), though the relationship between adiponectin and aldosterone was not demonstrated in this model.

**Table 3:** **Multiple linear regression analyses of the association of adiponectin and aldosterone using the stepwise variable selection procedure**

	**Adiponectin**		**Aldosterone**	
**Covariate**	**β ± SE**	**P=**	**β ± SE**	**P=**
**Age (per 5 years)**	0.445 ± 0.106	**<0.001**	−7.542 ± 2.218	**0.001**
**Waist (per 10 cm)**	−1.418 ± 0.283	**<0.001**	-	**-**
**Body fat (per 5%)**	-	**-**	14.329 ± 3.702	**<0.001**
**SBP (per 10 mmHg)**	0.236 ± 0.103	**0.023**	-	-
**IFG + DM (yes vs. no)**	−1.812 ± 0.597	**0.003**	-	**-**
**AC sugar (mM)**	-	-	0.487 ± 0.124	**<0.001**
**HDL-C (mM)**	4.512 ± 0.505	**<0.001**	-	**-**
**CRE (per μmol/L)**	-	-	0.039 ± 0.008	**<0.001**
**Adiponectin (μg/mL)**	-	-	−0.019 ± 0.025	0.436
**Aldosterone (per 100 pmol/L)**	−0.124 ± .0.065	**0.059**	-	**-**
**R** ^**2**^	0.239		0.104	

The abbreviations are the same as those in Table 1. Those p values < 0.05 were in bold form.

In order take into account possible confounding by medications being taken by the subjects, we further subcategorized our 556 subjects into whether they were taking anti-diabetic medications (n = 28) and anti-hypertension medications (89). We included these drugs as independent variables during the stepwise linear regression and found that they did not significantly affect our results. Additionally, we also tried excluding these subjects from our analysis and found our results to remain unchanged.

## Discussion

In this study we observed a rise in blood aldosterone levels along with a fall in blood adiponectin levels in the subjects with MetS and IR, though there was no strong independent association between these two factors. Adiponectin appeared to decrease along with the presence each MetS criterion met in our subjects, whereas aldosterone levels were only significantly higher in those subjects with MetS, i.e., those meeting three or more the criteria.

### Aldosterone and aldosterone vs. body fat

The links we found between adiponectin and greater waist circumference, impaired fasting glucose and diabetes were expected, because adiponectin is mainly synthesized and secreted by adipose tissue [27,28]. Adiponectin has also been found to enhance glucose utilization [29]. However, we also found a link between aldosterone and body fat, which is consistent with findings from two previous studies [20,30]. One recent study investigating gene expression of the steroidogenic enzymes in human adipose tissue has suggested that *de novo* biosynthesis of aldosterone in adipose tissue is not possible due the lack of key enzymes, such as aldosterone synthase [31]. However, human adipose tissue has been found to express angiotensinogen and ACE [32,33]. Therefore, the link we found between aldosterone and body fat may be at least in part a result effect of angiotensin, though further investigations are needed to fully define the exact mechanism behind this link between the two.

### Aldosterone and insulin resistance

Blood aldosterone levels showed a significant correlation with the IR index obtained by HOMA in the current study, similar to our results found for adiponectin. The current theories regarding the effects of aldosterone on glucose metabolism primarily focus on IR induced by inflammation, oxidative stress, hypokalemia and disturbance of insulin signaling and hepatic gluconeogenesis [20,34]. However, after detailed analysis of the adipose tissues from subjects with primary aldosteronism, Urbanet *et al*. concluded that IR in primary aldosteronism might occur in body compartments other than the adipose tissue [14]. Aldosterone may affect glucose metabolism in non-adipose tissues, including the islet *β*-cell function [34,35]. Some conditions, like obstructive sleep apnea-hypopnea syndrome (OSAHS) have been associated with metabolic syndrome [36]. Barceló *et al.* found a link between sleep apnea and aldosterone levels and reported those levels to be reduced by continuous positive airway pressure (CPAP) therapy after 12 months of treatment [37]. Kamide, in a cell culture study, reported that insulin increased the expression of angiotensinogen and angiotensin II [38], thus it may be possible that cardiovascular events in subjects with metabolic syndrome could be reduced with the use of agents that block RAAS. Cherney *et al.* reported that treatment with empaglifozin, a sodium glucose cotransport-2 (SGLT2) inhibitor, resulted in a decline in arterial stiffness in young subjects with type 1 diabetes and that the treatment induced a modest increase in plasma aldosterone levels [39]. The authors of that study proposed that these results could be related to the fact that SGLT2 inhibitor can cause a loss in body weight loss and a decrease in insulin secretion. Remírez *et al.* found eplerenone, an aldosterone antagonist, able to decrease hyperlipidemia, insulin resistance, myocardial free fatty acid uptake, and the accumulation of reactive oxygen species in an animal model [40]. However, the exact mechanism through which aldosterone modulates glucose tolerance and mediates the effects of MetS remains elusive, and thus merits further investigation.

### Negative relationship between adiponectin and aldosterone

The results of our regression model show a negative relationship between adiponectin and aldosterone, although this finding was of borderline significance. Aldosterone plays important roles in blood pressure regulation, and an inverse relationship between adiponectin and blood pressure has been reported previously in a study which also found that human adipocytes produced mineralocorticoid-stimulating factors that increased adrenal aldosterone secretion [41]. Increased aldosterone levels might then suppress expression of adiponectin protein through the mineralocorticoid receptor, as well as the glucocorticoid receptor. We postulate that increased adiposity or obesity directly decreases adiponectin synthesis in adipose tissue, and indirectly increases aldosterone production from the adrenal gland, probably secondary to increased angiotensin production from the adipose tissues. The results of the current study suggest that hypoadiponectinemia and hyperaldosteronism may work together to contribute to the development of glucose intolerance.

#### Limitation

This study has some limitations. One limitation is that it was an association study, and thus the cause-effect relationships cannot be inferred. Another limitation is that we used only women as study subjects. The observations reported in this study might thus not be generalized to men.

## Conclusions

Higher blood aldosterone levels and lower blood adiponectin levels were found in the female subjects with MetS and IR. Aldosterone levels were independently related to body fat % and fasting plasma glucose. However, we found no independent relationship between aldosterone and adiponectin after adjustment of various anthropometric and metabolic factors.

## Abbreviations

AECI: Angiotensin converting enzyme inhibitors
ARB: Angiotensin 2 receptor 1 blockers
CPAP: Continuous positive airway pressure
BMI: Body mass index
DM: Diabetes mellitus
HDL-C: High-density lipoprotein cholesterol
HOMA: Homeostasis model of assessment
HOMA-IR: Homeostasis model of assessment insulin resistance index
IR: Insulin resistance
LDL-C: Low-density lipoprotein cholesterol
mM: mmol/L
MetS: Metabolic syndrome
OSAHS: Obstructive sleep apnea-hypopnea syndrome
RAAS: Renin-angiotensin-aldosterone system
SGLT2: Sodium glucose cotransport-2
T-Chol: Total cholesterol
TG: Triglycerides
UA: Uric acid
WC: Waist circumference
